# Economic shocks and health resilience: lessons from the Russian Federation

**DOI:** 10.1093/pubmed/fdv166

**Published:** 2015-11-17

**Authors:** Vladimir S. Gordeev, Yevgeniy Goryakin, Martin McKee, David Stuckler, Bayard Roberts

**Affiliations:** 1 ECOHOST—Centre for Health and Social Change, London School of Hygiene and Tropical Medicine, London WC1H 9SH, UK; 2 Department of Health Services Research, CAPHRI, Maastricht University, Maastricht, The Netherlands; 3 Norwich Medical School, University of East Anglia, Norwich NR4 7TJ, UK; 4 Department of Sociology, Oxford University, Oxford OX1 3UQ, UK

**Keywords:** economic shock, health resilience, job loss, the Russian Federation, unemployment

## Abstract

**Background:**

Despite extensive research on determinants of health, there is much less information on factors protecting health among those exposed to economic shocks. Using longitudinal data from the Russian Federation in the post-Soviet period, we examined individual-level factors that enhance resilience of health to economic shocks.

**Methods:**

Logistic regression analysed factors associated with good self-assessed health (SAH) and health resilience, using pooled samples from the Russia Longitudinal Monitoring Survey-Higher School of Economics (1994–2012).

**Results:**

The general population consistently reported ‘average’ SAH, indicating almost invariant trends over the years. Male gender was the strongest predictor of good SAH and health resilience. Other factors positively associated with good SAH were age, higher education, employment, residing in rural areas, living in a larger and/or non-poor household. Among unemployed and those remaining unemployed, residing in rural areas, living in a larger and/or non-poor household remained the strongest predictors of good SAH and health resilience. These same factors were also important for males with recent job loss.

**Conclusions:**

Several factors predicting good SAH in the general population also influence health resilience factors among those remaining unemployed and experiencing a job loss. Such factors help to identify those most vulnerable and aid targeting assistance during economic crises.

## Introduction

Recent years have seen increasing research on the impact of economic crises on health.^1–3^ However, in every crisis, some groups fare worse than others. The concept of resilience refers to the ability of an individual, household, community or an entire system to withstand the negative impact of the stressor (e.g. an economic crisis). Consequently, while much research on such crises has focussed on those who suffer adverse health effects, there are also some who, despite similar shocks, do not do so or even demonstrate positive adaptation to adversity.^4–6^ Although, *prima facie*, it is likely that resilience and vulnerability vary according to the physical, psychosocial and economic characteristics of those exposed to the stressor, the concept of resilience varies across different disciplines so there is no single agreed definition.^4,7–11^ We have been able to identify a study that attempted to create a framework for assessing resilience of health systems in an economic crisis.^12^ However, although there is extensive research on vulnerability within the literature on determinants of health, our recent systematic review found that there is much less on factors protecting health and well-being, i.e. the determinants of resilience.^13^ This is unfortunate, because an understanding of why some individuals cope well and maintain good health despite experiencing continuing hardship or economic shocks may provide a valuable additional perspective.^14^ Such work could help reduce or prevent the development of adverse health outcomes.

In this paper, we look for factors that can enhance health resilience of individuals experiencing economic shocks, using a quantitative case study of the Russian Federation in the post-Soviet period. Following the radical, market-oriented reform along the lines of ‘shock therapy’ starting from early 1990s, the economy of the Russian Federation experienced several further economic crises (i.e. in 1998 and 2008–2009, following the worldwide recession). One could expect that severe changes in the economy could directly affect a population's health, making maintaining good health a challenge. The unemployed, in particular, would be expected to suffer the most during times of financial hardships.^15–18^ The aim of our paper is to identify factors associated with health resilience in Russia since 1994, specifically focussing on those who remain unemployed and those who lose jobs.

## Methods

### Data

**Fig. 1:**
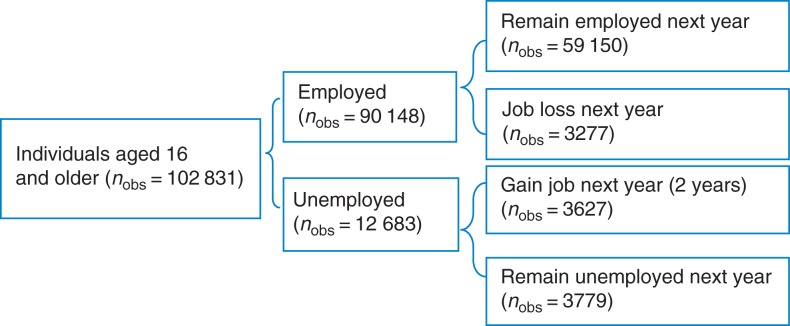
Overview of the samples. Source: Author's analysis based on RLMS-HSE. The data for 1997 and 1999 are not available.

Data were obtained from the Russia Longitudinal Monitoring Survey-Higher School of Economics (RLMS-HSE).^19^ The RLMS-HSE survey is a nationally representative survey designed to monitor the effects of Russian reforms on the health and economic welfare of households and individuals in the Russian Federation. It uses face-to-face interviews; more details may be found at http://www.cpc.unc.edu/projects/rlms-hse. For the analysis, we used the individual- and household-level data from Rounds 5–21 (1994–2012), although no rounds were conducted in 1997 and 1999. Data from Rounds 1–4 were sampled in a different way and are usually excluded from time series analyses. We used three samples in this study. The first sample includes all working-age individuals (defined according to Russian legislation), aged 16–60 years for males and 16–55 years for females (102 831 observations of 24 975 individuals) (Fig. 1). Using the panel structure of the data, the second and the third samples were restricted to those individuals present in at least two consecutive waves. The second is a pooled sample of individuals who remained unemployed in two consecutive years (3789 observations, 1900 individuals). The rationale for this sample is to identify the determinants of maintaining good SAH (or even improve from bad to good SAH), despite experiencing the economic hardship of remaining unemployed. The third sample comprised individuals who were employed in Year 1 but lost their job by Year 2 (3277 observations, 2705 individuals). This sample provides information on the factors that enable individuals to be resilient, in terms of SAH, despite the shock of job loss.

### Measures

#### Self-assessed health

SAH is among the most frequently assessed global measure of health, widely used in epidemiological studies and has been shown to predict mortality in diverse settings.^20,21^ In the RLMS-HSE data, SAH is measured using the question ‘How would you evaluate your health?’ on a five-point scale ranging from very bad, bad, average-not good, but not bad, good to very good. We dichotomized the outcome variable into ‘good’ (average, very good and good) and ‘bad’ SAH (very bad, bad), following previous studies with the RLMS-HSE^22,23^ and other Russian studies.^24–26^ We also constructed a variable measuring change in each individual's SAH, based on the difference in SAH in the current period (*t*), compared with the preceding period (*t*− 1). Should SAH have improved from bad in *t*− 1 to good in period *t*, or remained good in both *t* and *t*− 1 periods, the outcome variable was set as equal to 1 and otherwise 0.

#### Employment

An individual was classified as being unemployed if their present primary occupation was ‘temporarily not employed for other reasons (other than being student, health sickness, maternity leave retirement, etc.) and looking for a job’, as formulated in the questionnaire. Following the official definition of unemployment by International Labour Organization,^27^ this category excluded economically inactive individuals, i.e. those currently unemployed, but not looking for work. Those experiencing job loss and who remained unemployed during consecutive years *t*− 1 and *t* were identified.

#### Demographic, health behaviour and socioeconomic variables

To account for the non-linear relationship of SAH with age, age squared and cubed variables were created. Education was dichotomized into two categories: high (higher level education) and middle–low (complete secondary and incomplete secondary or primary). Area of residence was dichotomized into urban (regional centre, city and a category termed посёлок городского типа, meaning semi-rural settlement but with access to urban facilities) and rural. Household size captured the number of dwellers in each household. An asset score and a poverty measure captured the material and financial well-being of the household. The asset score was derived by summing a number of selected household goods (car, washing machine, videotape recorder, refrigerator, dacha—a country cottage or allotment) that loaded onto a single factor using the principal component analysis and formed a single continuous variable (0–5). This measure is similar to that used by Perlman and Bobak,^23^ with a refrigerator replacing a colour television. Each household belonged to one of five all-Russian poverty categories (with the 5th being the richest) as defined by Popkin *et al*.^28^ Prior to the analysis, the bivariate correlation between the asset score indicator and the poverty groups indicator was measured and found to be small (Pearson's correlation coefficient <0.3). This allowed us to use both variables in the regression analysis, avoiding the possible multicollinearity problem. The socioeconomic characteristics of the samples are summarized in Table 1. Respondents were classified as current smokers or non-smokers (either ex-smokers or never-smokers). The reported quantities of consumed alcohol within the last 30 days (beer, wine, fortified wine, vodka or home-distilled spirits) were converted into grams of pure alcohol based on conversion factors used in previous studies.^29,30^ We used the WHO definition of episodic heavy drinking as consuming >60 g of pure alcohol per drinking session^31^ to identify heavy drinkers.

**Table 1:** Distribution of variables among working-age respondents aged 16 and over (up to 55 years for women, 60 years for men)

*Characteristics*	*1994*	*2000*	*2006*	*2012*
Individual level				
Age (years), mean ± SD	37.32 ± 10.24	37.1 ± 10.47	37.09 ± 10.79	37.89 ± 10.59
Female 16–60 (*n*, %)	2169 (46.5)	2225 (48.5)	3127 (48.2)	4461 (47.7)
Male 16–55 (*n*, %)	2491 (53.5)	2362 (51.5)	3361 (51.8)	4885 (52.3)
Education				
Middle and low (*n*, %)	3690 (79.2)	3704 (80.7)	4980 (76.8)	6594 (70.6)
High (*n*, %)	970 (20.8)	883 (19.3)	1507 (23.2)	2752 (29.4)
Unemployed, yes (*n*, %)	567 (12.2)	732 (16)	829 (12.8)	822 (8.8)
SAH, mean ± SD	3.23 ± 0.64	3.29 ± 0.64	3.34 ± 0.61	3.44 ± 0.6
SAH				
Good (average to very good) (*n*, %)	4266 (91.5)	4268 (93)	6177 (95.2)	9039 (96.7)
Bad (very bad to bad) (*n*, %)	394 (8.5)	319 (7)	311 (4.8)	3073.3 (3.3)
Rural, yes (*n*, %)	1048 (22.5)	1237 (27)	1667 (25.7)	2245 (24)
Household level				
Household size, mean ± SD	3.58 ± 1.32	3.56 ± 1.45	3.5 ± 1.48	3.54 ± 1.54
Income per capita, rubles, mean ± SD	5224.55 ± 6347.15	3706.9 ± 4583.68	7675.48 ± 9442.15	11 074.22 ± 16 706.3
Asset score, mean ± SD	2.56 ± 1.05	2.88 ± 1.18	3.50 ± 1.35	3.54 ± 1.51
Poverty groups				
Poverty Group 1 (most poor) (*n*, %)	762 (16.3%)	805 (17.5%)	211 (3.3%)	77 (0.8%)
Poverty Group 2 (*n*, %)	1221 (28.6%)	1233 (26.9%)	471 (7.3%)	159 (1.7%)
Poverty Group 3 (*n*, %)	946 (20.3%)	972 (21.2%)	746 (11.5%)	322 (3.5%)
Poverty Group 4 (*n*, %)	615 (13.2%)	660 (14.4%)	891 (13.7%)	711 (7.6%)
Poverty Group 5 (most rich) (*n*, %)	1005 (21.6%)	917 (20.0%)	4167 (64.2%)	8077 (86.4%)
*n* respondents	4660	4587	6488	9346

The data for 1997 and 1999 are not available.

Source: Author's analysis of data from RLMS-HSE.

### Analyses

To measure health resilience, we adapted the following definition: ‘Resilience is the capability of individuals and systems (families, groups and communities) to cope successfully in the face of significant adversity or risk. This capability develops and changes over time, is enhanced by protective factors within the individual/system and the environment and contributes to the maintenance or enhancement of health’.^32^ Applying this definition to health resilience, we defined individuals as being resilient if their SAH either did not deteriorate or improve despite experiencing a shock or prolonged hardship (losing their job or being unemployed). The relationship between the outcome and independent variables that could potentially enhance or diminish individual's health resilience was assessed by means of descriptive and regression analyses, using STATA 14. In our analysis, we used the following model:

$$\text{logit}\{\text{prob}(\text{SA}{\text{H}}_{ij}=1)\;\}=\beta_{0}+\beta_{1}\text{SE}{\text{S}}_{ij}+r,\text{where}$$

SES*_ij_* is a vector of demographic and socio-demographic variables (gender, age, age^2^, age^3^, education level, area of residence, household size, household asset score and household poverty group); *β*_0_ is the constant term; *β*_1_ is a vector of parameters of interest; *r* is the error term. Several competing models were estimated to identify determinants of SAH. The best-fitting models were selected and are presented in the Results section.

## Results

**Fig. 2:**
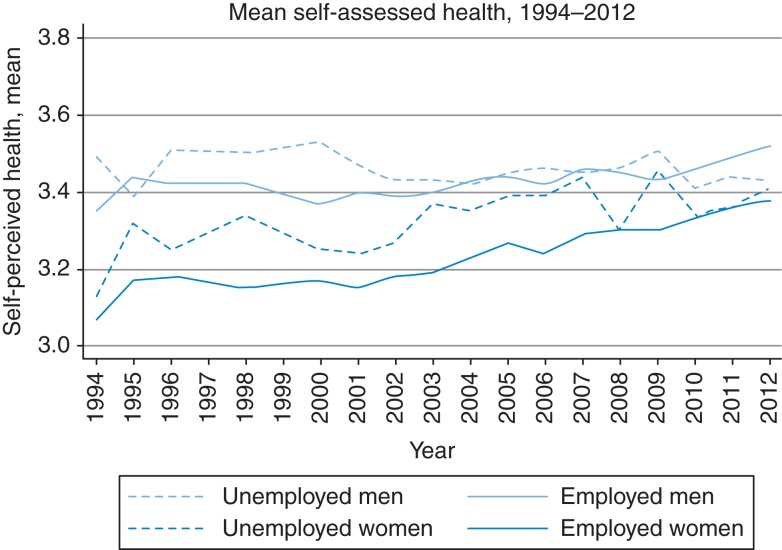
Change in self-assessed health among unemployed and employed. Source: Author's analysis of data from RLMS-HSE. The data for 1997 and 1999 are not available. The mean SAH among those unemployed seems to be higher compared with their employed counterparts, although in most periods the difference is not significant. This is most likely, because they are younger. After adjustment for individual- and household-level characteristics in the regression analyses, as shown in Table 2, ‘being employed’ status is a strong predictor of SAH.

Figure 2 shows trends in mean SAH over time for the general population, stratified by employment and gender. Overall, SAH improved over the study period (1994–2012) among those employed, for both genders, particularly from 2001 onwards. The mean values of SAH are higher among both employed and unemployed males than among women. Additional analysis (not shown) revealed that over the last two decades ∼55–60% of individuals in the general population consistently reported ‘average’ SAH, with almost invariant trends over the years.

**Table 2:** Factors associated with good health status in the general population (part A) and among unemployed people (part B) (1994–2012)

*Good health*	*Both genders*			*Female*			*Males*		
	*OR*	*95% CI*	**P* > *z**	*OR*	*95% CI*	**P* > *z**	*OR*	*95% CI*	**P* > *z**
General population (part A)									
Individual level									
Age	1.192	1.027–1.383	0.021	1.111	0.876–1.408	0.386	1.376	1.121–1.689	0.002
Age^2^	0.994	0.990–0.998	0.005	0.996	0.989–1.002	0.193	0.991	0.986–0.996	0.001
Age^3^	1.000	1.000–1.000	0.015	1.000	1.000–1.000	0.248	1.000	1.000–1.000	0.004
Male gender	2.018	0.444–0.553	0.000	—			—		
Higher education, yes	1.299	1.144–1.474	0.000	1.329	1.126–1.568	0.001	1.264	1.038–1.539	0.02
Household level									
Rural area	1.14	0.999–1.298	0.05	1.04	0.867–1.246	0.67	1.273	1.057–1.538	0.012
Employment, yes	1.508	1.344–1.691	0.000	1.439	1.225–1.692	0.000	1.59	1.351–1.872	0.000
Household size	1.129	1.092–1.167	0.000	1.137	1.087–1.190	0.000	1.121	1.068–1.176	0.000
Asset score 0–5	1.028	0.993–1.065	0.12	1.01	0.963–1.058	0.689	1.055	1.001–1.111	0.047
Poverty Group 1 (most poor)	Ref			Ref			Ref		
Poverty Group 2	1.117	0.968–1.288	0.131	1.216	1.003–1.474	0.047	1.003	0.81–1.243	0.975
Poverty Group 3	1.255	1.083–1.454	0.003	1.276	1.048–1.553	0.015	1.238	0.989–1.549	0.062
Poverty Group 4	1.375	1.180–1.603	0.000	1.401	1.143–1.717	0.001	1.351	1.069–1.707	0.012
Poverty Group 5 (richest)	2.118	1.852–2.423	0.000	2.308	1.924–2.768	0.000	1.874	1.534–2.289	0.000
	*n*_obs_ = 102 831; *n*_ind_ = 24 975			*n*_obs_ = 49 486; *n*_ind_ = 11 912			*n*_obs_ = 53 345; *n*_ind_ = 13 063		
Unemployed individuals only (part B)									
Individual level									
Age	1.194	0.865–1.649	0.281	2.214	1.235–3.970	0.008	0.92	0.600–1.410	0.701
Age^2^	0.993	0.985–1.002	0.149	0.975	0.959–0.992	0.004	1.001	0.989–1.012	0.907
Age^3^	1.000	1.000–1.000	0.178	1.000	1.000–1.000	0.004	1.000	1.000–1.000	0.852
Male gender	1.742	1.383–2.193	0.000	—			—		
Higher education, yes	1.081	0.768–1.521	0.655	1.235	0.734–2.078	0.427	0.976	0.615–1.55	0.918
Household level									
Rural area	1.627	1.261–2.098	0.000	1.496	1.004–2.228	0.048	1.791	1.281–2.505	0.001
Household size	1.148	1.071–1.231	0.000	1.286	1.140–1.449	0.000	1.07	0.982–1.166	0.122
Asset score 0–5	1.072	0.986–1.165	0.101	1.026	0.901–1.168	0.696	1.117	1.001–1.246	0.049
Poverty Group 1 (most poor)	Ref			Ref			Ref		
Poverty Group 2	1.007	0.758–1.339	0.962	0.955	0.614–1.486	0.839	1.02	0.700–1.486	0.916
Poverty Group 3	1.127	0.824–1.540	0.455	1.121	0.692–1.815	0.644	1.122	0.740–1.700	0.589
Poverty Group 4	1.131	0.804–1.590	0.480	1.116	0.662–1.881	0.681	1.102	0.699–1.737	0.675
Poverty Group 5 (richest)	1.412	1.060–1.880	0.018	1.582	1.013–2.471	0.044	1.274	0.871–1.863	0.212
	*n*_obs_ = 12 683; *n*_ind_ = 6604			*n*_obs_ = 5357; *n*_ind_ = 3019			*n*_obs_ = 7326; *n*_ind_ = 3585		

The data for 1997 and 1999 are not available. For all models, Prob > *χ*^2^ = 0.000.

Source: Author's analysis of data from RLMS-HSE.

Table 2 (part A) presents the results of the logistic regression of determinants of good SAH in the general population. Among males, good health was positively associated with age, being better educated, being employed, living in a larger household, a non-poor one and residing in a rural area. Among females, the pattern was similar, except that significantly better health began to be seen at lower poverty levels. When restricting the sample to those unemployed (Table 2, part B), among males, only living in a rural area and in a wealthier household were significantly associated with better health. Among females, age, residing in a rural area, living in a larger and wealthiest household were significant.

We now move from the static determinants of health to look at the determinants of health resilience (maintaining or improving SAH). Table 3 shows the change in SAH over consecutive waves in males and females who remain in employment, remain unemployed, gain employment or lose employment. We then look at the factors associated with health resilience in individuals who remained unemployed and thus experienced continuing economic hardship (Table 4 part A). Among males, the only significant factors were larger household size and not being among the poorest group. In females, the only factor was larger household size. When looking at the determinants of health resilience among individuals who experienced job loss (Table 4 part B), no factors were significant in females but, among males, living in a larger household and residing in a rural area were significantly associated with persistent good or improving health. The association of smoking and heavy drinking with SAH in both models was insignificant in both sexes (at *P* ≤ 0.05 level).

**Table 3:** Change in SAH over consecutive waves in males and females who remain in employment, remain unemployed, gain employment or loose employment

*Self-perceived health, mean ± SD*						
*Time period*	**t**	**t* + 1*	**t**	**t* + 1*	**t**	**t* + 1*
*Group*	*Women*		*Men*		*Overall*	
Employed in *t* and *t* + 1	3.23 ± 0.59	3.23 ± 0.57	3.42 ± 0.59	3.41 ± 0.59	3.33 ± 0.59	3.32 ± 0.59
Unemployed in *t* and *t* + 1	3.36 ± 0.67	3.34 ± 0.68	3.46 ± 0.69	3.45 ± 0.69	3.42 ± 0.68	3.41 ± 0.69
Employed in *t*, unemployed in *t* + 1	3.27 ± 0.63	3.26 ± 0.64	3.47 ± 0.65	3.42 ± 0.64	3.40 ± 0.65	3.36 ± 0.65
Unemployed in *t*, employed in *t* + 1	3.31 ± 0.64	3.34 ± 0.59	3.50 ± 0.65	3.49 ± 0.64	3.42 ± 0.65	3.43 ± 0.63

See comments beneath Fig. 2.

Source: Author's analysis of data from RLMS-HSE. The data for 1997 and 1999 are not available.

**Table 4:** Factors associated with a maintained good health or improved to good health status in those remaining unemployed over two consecutive waves and in those who lost job recently (1994–2012)

*Maintained good/improved to good health*	*Both genders*			*Female*			*Male*		
	*OR*	*95% CI*	**P* > *z**	*OR*	*95% CI*	**P* > *z**	*OR*	*95% CI*	**P* > *z**
Continuous unemployment (remaining unemployed over two consecutive waves) (part A)									
Individual level									
Age	1.460	0.763–2.795	0.253	2.389	0.687–8.309	0.171	1.263	0.556–2.869	0.576
Age^2^	0.987	0.969–1.005	0.161	0.972	0.937–1.008	0.130	0.992	0.970–1.014	0.468
Age^3^	1.000	1.000–1.000	0.151	1.000	1.000–1.001	0.126	1.000	1.000–1.000	0.466
Male gender	1.681	1.079–2.617	0.022	—			—		
Higher education, yes	1.849	0.784–4.361	0.160	1.046	0.305–3.586	0.943	3.235	0.921–11.363	0.067
Household level									
Rural area	1.649	1.039–2.614	0.034	1.714	0.840–3.498	0.139	1.664	0.898–3.084	0.106
Household size	1.255	1.099–1.433	0.001	1.343	1.075–1.679	0.009	1.202	1.018–1.419	0.03
Asset score 0–5	1.099	0.937–1.288	0.245	1.138	0.889–1.457	0.306	1.077	0.871–1.331	0.494
Poverty Group 1 (most poor)	Ref			Ref			Ref		
Poverty Group 2	0.503	0.297–0.852	0.011	0.607	0.278–1.325	0.21	0.391	0.187–0.815	0.012
Poverty Group 3	0.810	0.443–1.482	0.495	1.858	0.683–5.057	0.225	0.451	0.201–1.010	0.053
Poverty Group 4	0.499	0.268–0.930	0.029	1.496	0.529–4.230	0.447	0.237	0.103–0.544	0.001
Poverty Group 5 (richest)	0.629	0.356–1.112	0.111	0.622	0.272–1.425	0.261	0.593	0.264–1.334	0.207
	*n*_obs_ = 3789; *n*_ind_ = 1900			*n*_obs_ = 1403; *n*_ind_ = 779			*n*_obs_ = 2386; *n*_ind_ = 1121		
Recent job loss (part B)									
Individual level									
Age	0.753	0.391–1.452	0.397	0.509	0.106–2.444	0.399	1.065	0.509–2.227	0.867
Age^2^	1.006	0.988–1.023	0.528	1.014	0.971–1.059	0.536	0.997	0.978–1.017	0.802
Age^3^	1.000	1.000–1.000	0.532	1.000	1.000–1.000	0.604	1.000	1.000–1.000	0.864
Male gender	1.624	1.160–2.273	0.005	—			—		
Higher education, yes	0.851	0.540–1.342	0.488	0.796	0.348–1.821	0.588	0.865	0.484–1.546	0.624
Household level									
Rural area	1.104	0.988–1.233	0.081	1.025	0.831–1.263	0.819	1.164	1.011–1.340	0.035
Household size	1.446	0.990–2.113	0.056	1.229	0.610–2.477	0.565	1.604	1.004–2.564	0.048
Asset score 0–5	0.968	0.847–1.106	0.632	1.026	0.796–1.324	0.841	0.938	0.795–1.106	0.445
Poverty Group 1 (most poor)	Ref			Ref			Ref		
Poverty Group 2	1.073	0.621–1.855	0.800	1.002	0.350–2.870	0.996	1.096	0.569–2.113	0.784
Poverty Group 3	1.212	0.680–2.159	0.514	0.947	0.313–2.862	0.923	1.326	0.661–2.662	0.427
Poverty Group 4	2.232	1.128–4.416	0.021	1.555	0.442–5.476	0.492	2.875	1.209–6.838	0.017
Poverty Group 5 (richest)	1.507	0.920–2.467	0.103	1.155	0.441–3.028	0.769	1.781	0.991–3.203	0.054
	*n*_obs_ = 3277; *n*_ind_ = 2705			*n*_obs_ = 1263; *n*_ind_ = 1109			*n*_obs_ = 2014; *n*_ind_ = 1596		

The data for 1997 and 1999 are not available. For all models Prob > *χ*^2^ = 0.000.

Source: Author's analysis of data from RLMS-HSE.

## Discussion

### Main findings of this study

We began by looking at changes in SAH over the last two decades in the Russian Federation, subsequently exploring the determinants of good health and resilience to prolonged hardship and economic shocks. The mean SAH improved among those employed, of both genders, but fluctuated among the unemployed, improving among unemployed females, but not males (although this may be due to large variance in the mean SAH in these two groups). Male gender was the strongest predictor of good SAH. Other predictors of good SAH were age, employment status, living in larger and wealthier (non-poor) households, as well as having a higher level of education. Among the unemployed, living in larger households remained important when reporting good SAH, as did residing in a rural area.

Our results show that over the last two decades, most individuals in the general population consistently reported ‘average’ SAH, indicating almost invariant trends over the years. Thus, it seems that reporting SAH as ‘average’, rather than ‘good’ or ‘very good’, became a widely accepted norm in Russian society. The dominance of ‘average’ levels of SAH reported by individuals is unsurprising and is consistent with previous observations in the 1990s when few men or women in most former communist countries reported good SAH (compared with their Western European counterparts), with Russians reporting among the worst SAH levels, alongside those in Belarus and Hungary.^22,33^

### What this study adds

One of the clearest findings is that both unemployed and employed women continue to report worse SAH than their male counterparts do. This is in line with all previous findings on SAH in Russia and studies in other countries.^34,35^ This has been linked to selective survival, as there is little difference between healthy life expectancy in Russian men and women,^35,36^ but there may also be some difference in SAH between men and women in Russia. Our study focussed solely on the working-age population, excluding pensioners. Consequently, it was not possible to compare our results with an earlier study^37^ that showed how, even though women tended to report poorer health than men up to middle age, men between 45 and 59 years old reported the same overall health status as women, gradually losing their initial advantage and reporting the worst health status as pensioners.^37^ Nicholson *et al.*^25^ also showed in a cross-sectional study among those aged 50 and over that SAH in older Russians reflects social exposures accumulated over the life course, with the differentials observed only partially explained by current social conditions. In an age-adjusted analysis, good SAH was associated with high contemporary social class, income and education in both genders and, for men, not being widowed.^25^ This once again underlines the necessity to account for the changing perception of the SAH construct by individuals over the life span, as well as the age gradient among different genders. However, as noted above, it is also important to consider the persisting wide gender gap in the life expectancy, with older males being ‘survivors’.

Employment, education and household material well-being were among other predictors of SAH, consistent with studies conducted in other countries.^15–18,34,38–42^ Higher education was associated with better SAH in the general population, but not among the unemployed. This is in line with previous findings that find that the level of education or educational capital could^22,23,25,43,44^ or could not be^20,35,45^ associated with better SAH in the Russian population. Indicators of poverty and asset scores were associated with good SAH. Previous studies showed that personal income, family income and material deprivation are among the strongest individual predictors of SAH in Russia,^22,23,25,35,44^ as well as other indicators of economic circumstances (economic difficulties during childhood, number economic difficulties during the past 12 months, changes in material well-being over 10 years).^20^

Residing in a rural area was another important factor associated with positive SAH (particularly among males), and this association appears stronger in the continually unemployed sample. We hypothesize that this is potentially due to the presence of a stronger informal social support network in rural areas, a higher level of social capital, somewhat less stressful living environment, as well as reliance on natural production (growing and storing own crops, gathering and foraging). However, the lack of relevant data in the RLMS-HSE dataset precludes testing these assumptions but these issues could be explored in future studies.

Living in larger households was protective, even after controlling for gender, education and wealth. This is consistent with studies showing that being married, as well as having children in the family act as protective factors for physical health^34^ and mental health (stress levels and happiness).^46,47^ It could be that, in larger families, people tend to report better SAH regardless of their employment status or other socioeconomic characteristics, due to greater social cohesion, support and feelings of security. However, we were unable to test this assumption.

### What is already known on this topic

The potential importance of social and human capital and income inequalities is supported by earlier studies suggesting that they could have a strong compositional effects on SAH, with contextual country specific measures possibly moderating the effect of compositional measures on SAH.^35,48,49^ Social capital elements (e.g. contact with neighbours; being a member of a trade union or a political organization or any other organization; social exclusion; reliance on anti-modern, market, informal networks) have been shown to be positively associated with good SAH.^20,35,43^ The contribution of social capital outside the family (e.g. visiting friends and acquaintances, membership of voluntary associations) to SAH seems to be particularly important for men, proving once again the necessity to account for different gender roles, socializing patterns, and the values embedded within them, in the Russian (and not only) society, since social capital provides access to different forms of resources, influences and support, but also imposes different obligations.^50^ Therefore, the concepts of social and human capital could be important in better understanding health resilience and should be included in future studies, subject to availability of such data.

### Limitations of this study

The limitations of this study are those common to any using self-reported and panel data: reporting bias; attrition in the individual and household longitudinal constituents reducing sample size of unemployed individuals, less precise model parameter estimations and reduced power; short recall period of 30 days (income, employment and SAH); data being collected only once a year, which does not allow us to monitor changes in SAH and work status occurring between two consecutive periods, leading to an imposed assumption of individuals staying in the same continuous reported state; exclusion of ‘invisible’ people that are even more likely to suffer from micro- and macro-economic shocks, including homeless people, undocumented immigrants and prisoners; underrepresentation of marginal and very rich people; and lack of variables on social capital and social cohesion. Another limitation of our study is that we could not ascertain causal relationships given the study design.

## Conclusions

We showed that several factors predicting good SAH in the general population also influence health resilience factors among those remaining unemployed and those who experience job loss. Such factors help to identify those most vulnerable and aid targeting assistance during economic crises. However, identifying factors that help individuals remain healthy despite adversity and shocks is only half the task. What remains to be studied are the exact mechanisms that underpin resilience, using both quantitative and qualitative data. This could be explored further using other longitudinal datasets (e.g. the German Socio-Economic Panel^51^ and the UK Understanding Society Study),^52^ especially due to recurrent nature of recent economic crises. The next step, should the data be available, is to explore casual relationships between shocks and adversity, factors promoting resilience, and SAH, which would strengthen evidence that can be translated into policy.

## Authors' contributions

V.S.G. drafted the paper together with input from Y.G., D.S., M.M. and B.R. V.S.G. conducted the statistical analysis with the input from Y.G., D.S., M.M. and B.R.

## Funding

This work was funded by the Economic and Social Research Council (grant ref: ES/K003496/1) (http://www.esrc.ac.uk/). DS is funded by ERC HRES 313590 and a Wellcome Trust Investigator Award. The funders had no role in study design, data collection and analysis, decision to publish or preparation of the manuscript.
