# Economic shocks and health resilience: lessons from the Russian Federation

**DOI:** 10.1093/pubmed/fdx084

**Published:** 2017-07-13

**Authors:** Vladimir S Gordeev, Yevgeniy Goryakin, Martin McKee, David Stuckler, Bayard Roberts

*J Public Health (Oxf)* 2016; 38 (4): e409–e418. doi: 10.1093/pubmed/fdv166

The funding information should have included the following statement:

DS is funded by ERC HRES 313590 and a Wellcome Trust Investigator Award.

The online article has been corrected.

